# Study of the Effect of Carbon Black Filling on the Mechanical Behavior of Rubber Hyper-Elasticity

**DOI:** 10.3390/ma16196561

**Published:** 2023-10-05

**Authors:** Zepeng Wang, Xiulong Yao, Fangru Hu, Chuanxiang Ma, Xinyan Li, Zhanli Miao, Junping Song, Lianxiang Ma, Wei Li

**Affiliations:** 1 College of Electromechanical Engineering, Qingdao University of Science and Technology, Qingdao 266061, China; 02546@qust.edu.cn (Z.W.); 4020030117@mails.qust.edu.cn (X.Y.); 4021030097@mails.qust.edu.cn (F.H.); 4021030113@mails.qust.edu.cn (C.M.); 4021030102@mails.qust.edu.cn (X.L.); 01887@qust.edu.cn (Z.M.); oldhorse@qust.edu.cn (L.M.); 2 Department of Energy Engineering, Zhejiang University, Hangzhou 310027, China

**Keywords:** natural rubber, Yeoh constitutive model, hyper-elastic, carbon black filling

## Abstract

We have particularly investigated the correlation law of the effect of different carbon black fillings on the hyper-elastic mechanical behavior of natural rubber by conducting uniaxial tensile tests over a wide range of deformations with different volume fractions of carbon black fillings (0%, 4.7%, 8.9%, 12.8%, 16.4%, 19.7%, 22.7% and 25.2%). The results show that the stress-strain curve for carbon black filled rubber increases with the amount of filling, meaning that the rubber gradually becomes “harder”. We explore the correlation between the carbon black filling volume and the parameters of the Yeoh constitutive model by examining the Yeoh constitutive model to characterize the hyper-elastic mechanical behavior of rubber with different carbon black fillings. A quantitative relationship between the material parameters and the carbon black filling volume in the Yeoh constitutive model is presented. A method for calculating the material parameters of the Yeoh constitutive model is developed, and it predicts the correlation between the hyper-elastic properties of rubber and the volume fraction of the carbon black filling.

## 1. Introduction

Carbon-black-filled rubber composites are widely used in sealing, noise reduction, vibration damping and transportation applications due to their high hardness, high elasticity and tear strength [1,2]. In addition, finite element analysis is an important means of evaluating their mechanical properties. When performing finite element analysis on rubber composites, the determination and selection of a suitable hyper-elastic constitutive model based on experimental data are fundamental and critical to the success or failure of the analysis. In order to reasonably describe the stress-strain relationship in rubber composites, different hyper-elastic constitutive models have been developed by the engineering and scientific communities. The stress-strain relationship of rubber materials is represented by the strain energy function [3]. The main hyper-elastic constitutive models of rubber are divided into two types: the image-only theoretical models based on the mechanics of continuous media and the statistical models based on molecular chain networks [4,5]. In order to study the theoretical modelling of the fundamental elastic and viscoelastic behavior of carbon-black-filled rubber, the Yeoh model was chosen by N. KOPROWSKI-THEISS for the reason that only uniaxial tensile tests were carried out. And the Yeoh model, as an intrinsic model of fundamental elasticity, is able to represent this non-linear behavior [6]. M.R. Mansouri has proposed an exponential framework of strain energy density functions for elastomers and soft biological tissues, inspired by the image-only Yeoh model. The model is simple, parametrically stable and provides more accurate predictions of the stress response of elastomers and soft tissues for a wide range of properties [7]. Zhao proposed a hyper-elasticity model for isotropic, incompressible types of rubber materials in order to examine the predictive capability of the new model. He identified the parameters of the new model, the Yeoh model and the Carroll model using experimental data for 8% vulcanized rubber and two different types of carbon-black-filled rubber, respectively [8]. Anand determined the important fracture parameters of the NR/BR blends using experimental and finite element modelling methods, where the Yeoh model was used to characterize the hyper-elastic mechanical behavior of the material [9]. El Yaagoubi compared the life prediction capabilities of the image-only based Yeoh model and the inelastic MORPH model, but the Yeoh model does not reproduce properties such as softening effects and hysteresis losses of the material [10]. Uday has successfully modelled the hyper-elastic properties of retreaded tire rubber using the finite element method. The Yeoh and Arruda-Boyce constitutive models demonstrated parametric stability and accuracy of fit, as measured by the accuracy of test results from fitted uniaxial and planar tensile experiments [11]. He Hong evaluated the ability of 85 hyper-elastic constitutive models to reproduce experimental data for unfilled and highly filled rubber nanocomposites. He found that the image-only based Yeoh model was a good fit for the highly filled HNBR [12]. It has been shown that the Yeoh model is a good description of the mechanical response of rubber hyper-elastic materials over a wide range of deformations and that it can be applied to a wide range of practical engineering problems.

In the previously mentioned investigations, the Yeoh constitutive model has been used to describe the relationship between the shear modulus of filled rubber and its deformation. However, the amount of reinforcing agent usually determines the mechanical response of the rubber hyper-elastic material. Therefore, it would be useful to be able to develop a hyper-elastic constitutive structure model using an already existing one. And it is worth exploring whether it can describe different carbon black fillings of rubber composites and also predict the mechanical characteristics of a range of different rubber formulations. The model can accurately reflect the hyper-elastic mechanical behavior of rubber composites with different carbon black contents and also predict the mechanical response of rubber composites in terms of carbon black fillings.

This article reflects the effect of carbon black fillings on the hyper-elastic mechanical characteristics of rubber through a combination of theoretical characterization and experimental analysis. The correlation between the hyper-elastic mechanical properties of carbon-black-filled rubber, the volume fraction of carbon black filling and the parameters of the Yeoh constitutive model is investigated in this paper. It can provide theoretical support and new ideas for predicting the stress-strain relationship of carbon-black-filled rubber in a wide range of deformations. Section 2 introduces the strain energy density function and the stress-strain relationship equation for the Yeoh constitutive model. Section 3 describes the manufacturing of experimental materials, experimental formulations and experimental equipment. Section 4 shows the stress-strain hyper-elastic mechanical behavior of pure natural rubber and rubber with different carbon black fillings over a wide range of deformation (strain of 150%). This not only reveals the correlation between the material parameters of the Yeoh model and the volume fraction of carbon black filling, but also verifies the predictive ability of the Yeoh model with an explicit volume fraction of carbon black filling parameters on the hyper-elastic mechanical behavior of rubbers with different carbon black fillings. Combining the relationship between the parameters of the Yeoh constitutive model and the volume fraction of carbon black filling, a Yeoh intrinsic model for explicitly characterizing the amount of carbon black filling is constructed. A summary analysis and conclusions are given at the end of the text.

## 2. Constitutive Models

The currently commonly used constitutive models for rubber hyper-elasticity can be broadly categorized into two types. One is molecular network models based on thermodynamic statistical theory. These models are used to examine the configurational entropy changes within molecular networks, which require relatively fewer model parameters to predict the hyper-elastic mechanical behavior under large strains. Examples of such models include the three-chain model [13], the four-chain model [14] and the Arruda-Boyce model [13]. The other type is phenomenological models based on continuum mechanics theory. These models are used to predict the elastic response of both unfilled and filled rubber under large strains. Examples include the Neo-Hookean model [15], the Mooney-Rivlin model [16] and the Yeoh model [17]. In this study, the Yeoh constitutive model was selected, as it is suitable for various deformations and particularly well-suited to uniaxial tension. The most commonly used form of the strain energy density function in the form of a deformation tensor invariant series was initially proposed by Rivlin [18], that is, the reduced polynomial model

(1) $$W=\sum_{i+j=1}^{N}C_{i}{}_{j}{\left(I_{1}-3\right)}^{i}(I_{2}-3{)}^{j}$$

After analyzing experimental data for a filled rubber material, Yeoh proposed a simplified polynomial strain energy function. The incompressible Yeoh model assumes that the strain energy function is a general polynomial of the first principal tensile invariant *I*_1_ only [15]. In the case of in the reduced polynomial model as expressed in Equation (1), it is exactly the Yeoh constitutive model, and its strain energy density function is as follows:

(2) $$W=C_{10}(I_{1}-3)+C_{20}{\left(I_{1}-3\right)}^{2}+C_{30}{\left(I_{1}-3\right)}^{3}$$

The Yeoh constitutive model can generate typical S-shaped stress-strain curves, which better capture the highly nonlinear mechanical characteristics of rubber materials in hyper-elasticity. The relationship between the nominal stress (*σ*) and draw ratio (*λ*) for the Yeoh constitutive model under uniaxial tension is as follows:

(3) $$\sigma =2(\lambda^{2}-\frac{1}{\lambda })\left[C_{10}+2C_{20}(\lambda^{2}+\frac{2}{\lambda }-3)+3C_{30}{\left(\lambda^{2}+\frac{2}{\lambda }-3\right)}^{2}\right]$$

where *C*_10_, *C*_20_, *C*_30_ are undetermined material model parameters that can be determined through uniaxial tensile tests. The variation trends of the parameters in the Yeoh constitutive model differ across different deformation stages. In the case of small deformations, *C*_10_ represents half of the initial shear modulus. During moderate deformations, *C*_20_ is generally a negative value, indicating the softening behavior of the material at this stage. *C*_30_ reflects the phenomenon where the material starts to stiffen again within a larger deformation range.

## 3. Experimental Setup

### 3.1. Experimental Materials

Eight different rubber specimens with varying carbon black contents were used in the experiments. The rubber matrix was composed of natural rubber, while the filler was carbon black N220. Among the eight rubber formulations, only the quantity of the carbon black filler differed. The eight rubber types (NR-0, NR-1, NR-2, NR-3, NR-4, NR-5, NR-6 and NR-7) had carbon black filling mass fractions of 0 phr, 10 phr, 20 phr, 30 phr, 40 phr, 50 phr, 60 phr and 70 phr, respectively. The names, specifications and manufacturers of the main raw materials used in the experiments are shown in Table 1. The rubber models and their formulations used in the experiments are presented in Table 2.

### 3.2. Sample Preparation and Test

The rubber mixing process was divided into two stages. First, natural rubber was pressed for 3 min on an S(X)−160A two-roll mill (Shanghai First Rubber Machinery Co., Ltd., Shanghai, China). The compressed natural rubber was then transferred to a Hakke torque rheometer. In sequence, zinc oxide, stearic acid, antioxidant 4020 and carbon black were added for the first-stage mixing, which continued for 10 min. When the temperature was reduced to 100 °C, the machine was stopped to discharge the material.

The second-stage mixing was performed on the two-roll mill. The roll shaft temperature was set to (60 ± 5) °C, and the gap between the two rolls was adjusted to 3–5 mm. After that, the material from the first-stage mixing was preheated and evenly wrapped on the rolls. Sulfur and accelerator NS were added, and the mixing continued until the material was fully uniform. At approximately 3/4 of the position of the mixing material, three cuttings were executed on both sides of the material using a knife. It was then shaped into five triangular packs and five roll packs. Following this, the roll gap was adjusted to 1.5 mm, and the material was thinly passed through the rolls seven times to ensure thorough mixing. The material was compressed to a thickness of 3–5 mm for sheet production, obtaining the CB/NR composite material. The CB/NR material was left undisturbed at room temperature (20 °C) for a minimum of 5 h before proceeding with vulcanization. Subsequently, the vulcanization properties were measured following GB/T 9869-2014 [19]. The sulfur vulcanization characteristics, including the vulcanization curve, minimum torque (ML), maximum torque (MH) and the optimum cure time (Tc90), were determined using a moving die rheometer. The vulcanization was carried out at 150 °C, 10 MPa pressure, and for an equivalent cure time (Tc90). When the temperature of the plate vulcanization press reached 150 °C, the rubber was placed into the mold for vulcanization. After vulcanization, the samples were cooled and allowed to sit for 12 h for subsequent performance characterization. According to ISO 37-2017 [20], Type 2 dumbbell specimens with a rubber thickness of 2 mm were prepared.

**Figure 1:**
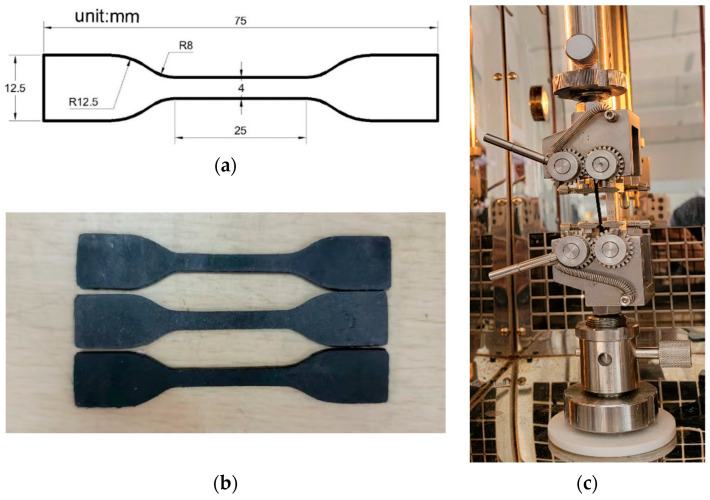
Dumbbell type 2 specimen size (**a**); dumbbell 2 rubber specimen (**b**); and double eccentric wheel fixture RA-4-1 (**c**).

**Figure 2:**
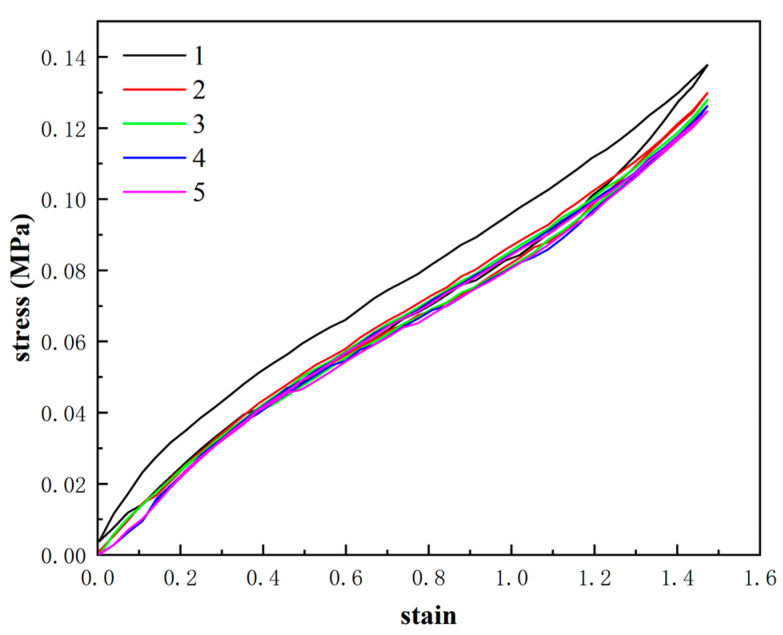
Stress-strain curve of modulated rubber specimen NR-0 at 290 K.

Under the condition of 290 K, a quasi-static uniaxial tensile test was conducted on the specimen shown in Figure 1a using a computer-controlled tensile testing machine (MTS CMT4104). Prior to the formal test, the Mullins effect [16] of the specimen should be eliminated. The Mullins effect, also known as the stress-softening effect, refers to the response when the produced stress of the non-deformed rubber material during the initial loading deformation is greater than that during subsequent loading deformations. While, when the strain exceeds the initial strain, the stress becomes greater than the initial stress and is unaffected by the initial deformation state. In order to eliminate the Mullins effect, the loading-unloading cycle was repeated five times at a tensile rate of 100 mm/min, and 150% elongation was chosen as the cyclic modulation strain. From Figure 2, it can be seen that the stress-strain curve stabilizes after 5 modulations of the rubber specimen. The modulation temperature was 290 K, and the fixture measurement distance was 25 mm. The purpose of modulation is to more accurately reproduce the stress state of tire rubber. During the uniaxial tensile testing, an RA-4-1 double eccentric wheels fixture was employed, which is a special tensile fixture for rubber, as shown in Figure 1c. After modulation, the rubber specimens were allowed to stand for 24 h to fully recover their elastic deformation and stabilize their properties. The experiment was repeated at least five times for each condition, and the average value was taken as the final experimental result.

## 4. Experimental Result and Discussion

### 4.1. The Uniaxial Tensile Tests Results

**Figure 3:**
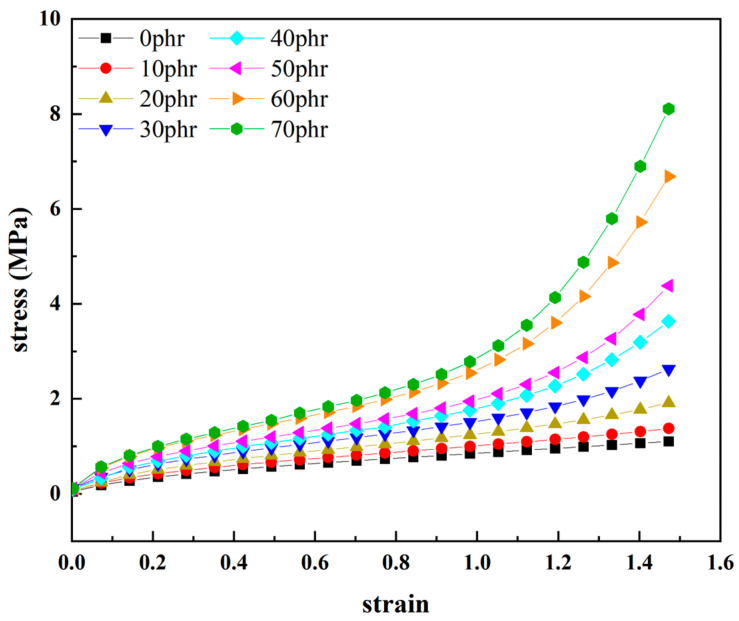
Uniaxial tensile stress-strain curves of natural rubber with different carbon black fillings.

Figure 3 illustrates the uniaxial tensile stress-strain curves of natural rubber with varying levels of carbon black filling content under significant deformation ranges (150% strain). From the figure, the stress-strain relationship of natural rubber increases overall with an increase in the carbon black filling content. This indicates that the addition of carbon black results in material stiffening due to its reinforcing effect, leading to an increase in the material’s stiffness modulus. As the carbon black filling content increases, the stress-strain relationship of natural rubber becomes steeper, evolving into a more pronounced “S”−shaped nonlinear characteristic. The greater the strain level, the more prominent the steepness of the curve. Although the carbon black filling content varies, the stress-strain relationship of natural rubber consistently exhibits a distinct “S”−shaped nonlinear characteristic. Moreover, as the carbon black filling content increases, the “S”−shaped nonlinear characteristic in the stress-strain relationship of natural rubber becomes even more pronounced. These results indicate that the filled rubber materials can exhibit different deformation behaviors at various deformation stages. Therefore, investigating the hyper-elastic properties of carbon-black-filled rubber under large deformations is of significant importance.

**Figure 4:**
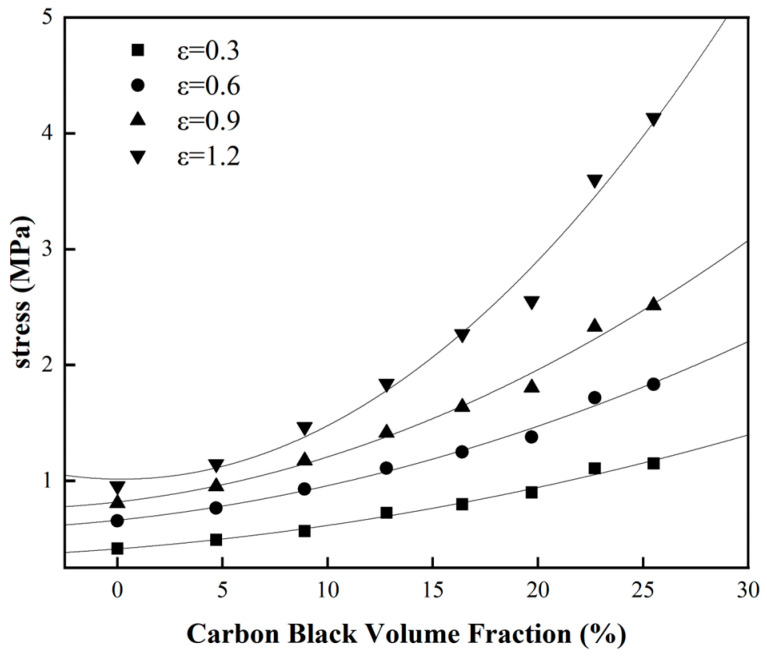
The variation trend of rubber stress with the volume fraction of CB under constant strain (*ε* is strain in the figure).

To further elucidate the correlation between the hyper-elastic mechanical properties of the natural rubber and carbon black filling, Figure 3 was processed to obtain the stress curve of natural rubber with different carbon black filling contents under different constant strains over the carbon black filling volume fraction, as shown in Figure 4. From Figure 4, it is evident that as the carbon black filling content in the rubber increases, the stress behavior of the rubber at different strains shows a clear trend of nonlinear growth. In other words, as the carbon black filling content increases, the rubber gradually becomes stiffer, and the reinforcing effect on the rubber becomes highly noticeable, manifesting as a distinct hyper-elastic response. Furthermore, as the carbon black filling content increases, the mechanical properties of the rubber are also enhanced, as indicated by the pronounced upward curvature at the right end of the curves in Figure 4.

In order to further quantify and analyze the reinforcing mechanism of the carbon black filling in the rubber matrix, Fukahori et al. [21] introduced the concept of the “stress amplification factor”. This concept aims to elucidate the reinforcing effect of the carbon black filling on the stress in rubber at a fixed strain, i.e.,

(4) $$\alpha =\sigma /\sigma_{0}$$

where *σ* represents the stress of the rubber composite material at a certain strain, and *σ*_0_ represents the stress of sulfur-cured natural rubber at the corresponding strain. From the definition in Equation (4), it can be understood that *α* reflects the extent to which the stress in the carbon-black-filled material is enhanced under a certain strain. In other words, it indicates the multiplier by which the stress in the carbon-black-filled rubber is amplified at a constant elongation ratio. This intuitively demonstrates the “stiffening” characteristic of the material due to the carbon black filling.

**Figure 5:**
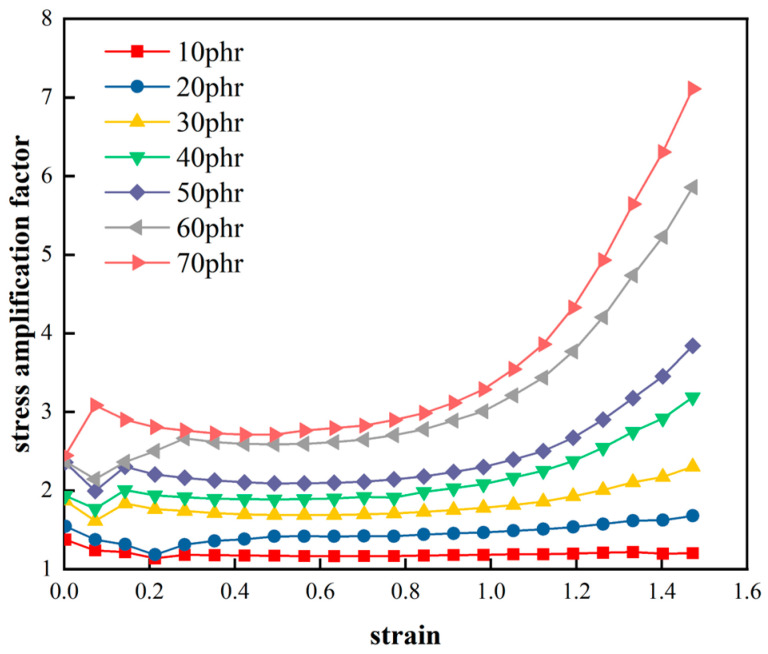
Stress amplification factor α versus strain of rubber with different CB filling ratios.

Using Equation (4), the variation curve of the “stress amplification factor” for filled rubber with respect to nominal strain can be obtained, as shown in Figure 5. From Figure 5, the “stress amplification factor” of natural rubber with different carbon black filling contents first decreases and then increases with an increase in the tensile length. This indicates that as the deformation level of the material increases within a larger deformation range, the phenomenon of stress enhancement with nonlinear variations becomes more pronounced. Moreover, as the number of carbon black fillings in the rubber increases, this “first decrease and then increase” nonlinear characteristic becomes more significant. This suggests that in the tensile process of carbon-black-filled rubber, different elongation ratios correspond to different enhancements and amplifications of the rubber’s hyper-elastic mechanical behavior. Additionally, the reinforcing effect of the rubber increases with an increase in the carbon black filling content.

Figure 5 also indicates that in the initial stages of small deformation, the amplification factor of materials with different carbon black filling contents is particularly unstable. This may be due to instrument errors introduced at the start of the measurements. Meanwhile, the “stress amplification factor” for different materials tended to stabilize at nominal strains ranging from 0.2 to 0.7. This suggests that during this deformation stage, the phenomena of stress enhancement and amplification became relatively constant, and as the tensile length continued to increase, the stress enhancement and amplification phenomena exhibited an exponential trend. Additionally, it was observed that the “stress amplification factor” *α* for all eight rubber samples stabilized at a strain of 0.3.

### 4.2. Discussion

The experimental results for rubber samples with different carbon black filling contents (NR-0, NR-10, NR-30, NR-50 and NR-70) were fitted and analyzed using the Yeoh constitutive model. In order to assess the goodness of fit of the model, the coefficient of determination *R*^2^ was calculated,

(5) $$RSS={\sum_{i=1}^{N}\left(P_{i}-\overset{\wedge }{P_{i}}\right)}^{2},TSS={\sum_{i=1}^{N}\left(P_{i}-\overset{-}{P_{i}}\right)}^{2},R^{2}=1-RSS/TSS$$

where *P_i_* represents the experimental values, ${\hat{P}}_{i}$ represents the model-fitted values, ${\bar{P}}_{i}$ *P_i_* represents the mean of the experimental values, and *N* represents the number of experimental data points used in the fitting process.

**Figure 6:**
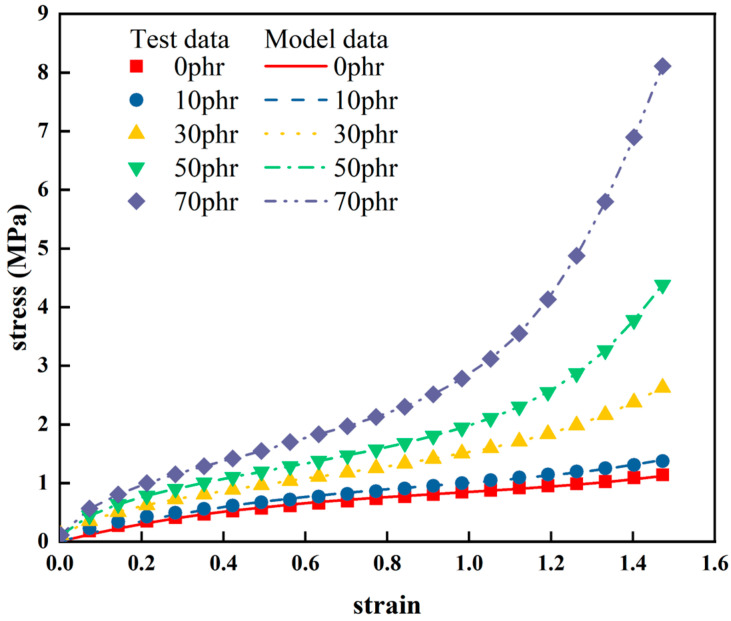
Fitting results of improved model for some rubbers with different CB fillings.

The research results are presented in Figure 6. Table 2 provides the Yeoh constitutive model parameters and coefficients of determination (*R*^2^). From Figure 6 and Table 3, it can be observed that the fitting curves for natural rubber with different carbon black filling contents closely match the experimental curves (with *R*^2^ values all above 0.9). This indicates that the Yeoh constitutive model can accurately characterize the hyper-elastic mechanical behavior of natural rubber under different carbon black filling contents. It demonstrates the robust generality of the Yeoh constitutive model in describing the hyper-elastic mechanical behavior of natural rubber.

The characterization results for natural rubber with different carbon black filling contents provided only the qualitative analysis of the model’s ability to represent the hyper-elastic mechanical properties of the filled rubber. They neither directly indicated the influence of carbon black filling content on model parameters, nor quantitatively described the relationship between the carbon black filling volume fraction and the hyper-elastic mechanical behavior. To address this issue, the macroscopic phenomenological theory was used to establish a quantitative relationship between the corresponding material parameters in the Yeoh constitutive model and the carbon black filling volume fraction.

**Figure 7:**
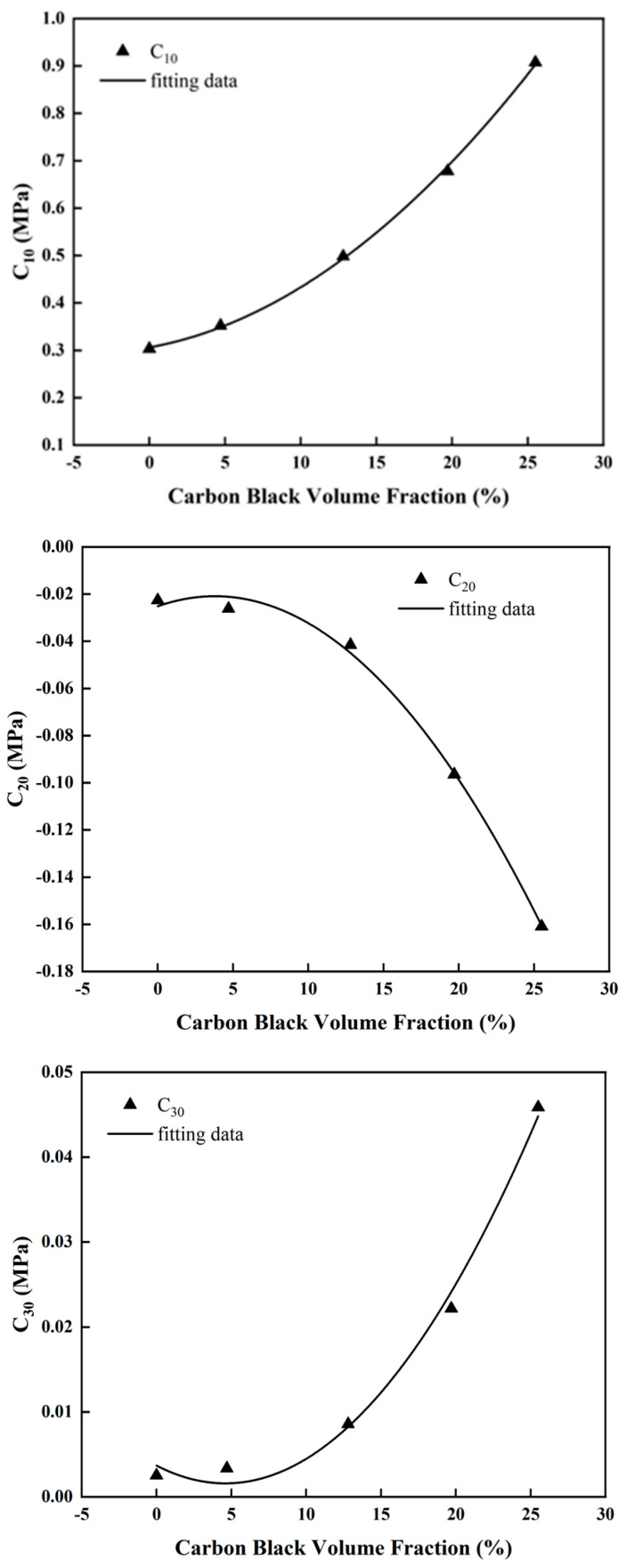
Variation trend of parameters of Yeoh model with CB volume fraction and the corresponding fitting curve.

Based on experimental stress-strain performance results for rubber samples with varying carbon black filling contents (NR-0, NR-10, NR-30, NR-50 and NR-70), fitting was performed for the material parameters *C*_10_, *C*_20_ and *C*_30_ in the Yeoh constitutive model, as depicted in Figure 7. Since the material parameter *C*_10_ represents half of the shear modulus at small strains, the rubber gradually stiffens with an increase in the carbon -black-filling volume fraction, resulting in a gradual increase in its shear modulus. As a result, the material parameter *C*_10_ increases with the rising carbon-black-filling volume fraction, whereas the material parameter *C*_20_ represents the softening phenomenon of the filled rubber under moderate deformation. A larger *C*_20_ indicates a more pronounced softening effect. Conversely, when *C*_20_ is smaller, the rubber is stiffer, indicating a higher carbon-black-filling volume fraction. Furthermore, material parameter *C*_30_ signifies the phenomenon where the material starts to stiffen again under large deformations. As the carbon black filling volume fraction increases, *C*_30_ also increases. The fitting results demonstrate that the material parameters *C*_10_, *C*_20_ and *C*_30_ in the Yeoh constitutive model all exhibit clear quadratic relationships with the carbon-black-filling volume fraction. Based on the analysis above, the quantitative relationship between the material parameters *C*_10_, *C*_20_ and *C*_30_, and the carbon-black-filling volume fraction can be expressed using quadratic functions, as shown in Equation (6).

(6) $$\left\{\begin{array}{l} C_{10}=A_{0}+A_{1}V+A_{2}V^{2}\\ C_{20}=B_{0}+B_{1}V+B_{2}V^{2}\\ C_{30}=C_{0}+C_{1}V+C_{2}V^{2} \end{array}\right.$$

where *V* represents the carbon-black-filling volume fraction in the rubber specimen, and *A*_0_, *A*_1_, *A*_2_, *B*_0_, *B*_1_, *B*_2_, *C*_0_, *C*_1_ and *C*_2_ are the fitting parameters to be determined. In this paper, they are referred to as the correlation characterization parameters for the carbon-black-filling volume fraction. The specific values of the material parameters *C*_10_, *C*_20_ and *C*_30_ have already been obtained through fitting, as shown in Table 3. By utilizing the Yeoh constitutive model fitting parameters from Table 3 and Equation (6), the characterization parameters for the carbon-black-filling volume fraction can be computed, as presented in Table 4.

Thus, the Yeoh instanton model for the filled volume fraction of apparent carbon black is obtained by combining Equation (7) with Equation (6).

(7) $$\left\{\begin{array}{l} W=C_{10}(I_{1}-3)+C_{20}{\left(I_{1}-3\right)}^{2}+C_{30}{\left(I_{1}-3\right)}^{3}\\ C_{10}=A_{0}+A_{1}V+A_{2}V^{2}\\ C_{20}=B_{0}+B_{1}V+B_{2}V^{2}\\ C_{30}=C_{0}+C_{1}V+C_{2}V^{2} \end{array}\right.$$

**Figure 8:**
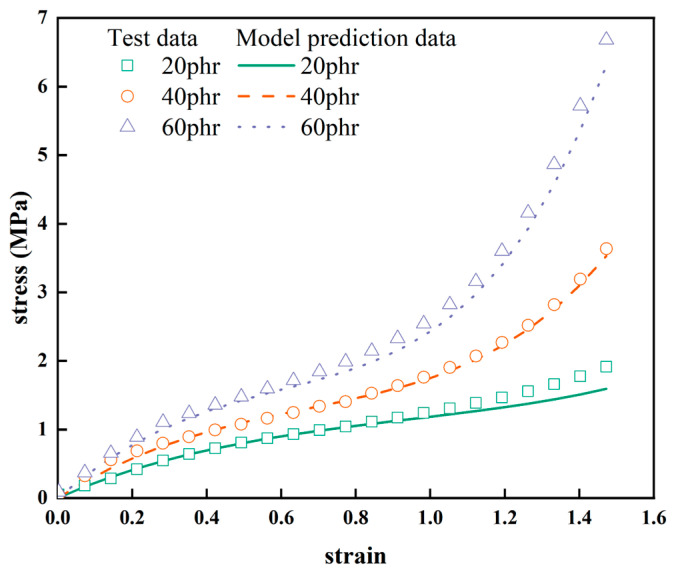
Predicted stress-strain curves by Yeoh model with apparent carbon-black-filling volume fraction.

Using Equation (7) and the correlation characterization parameters for the carbon-black-filling volume fraction in Table 3, the stress-strain curves for NR-2, NR-4, and NR-6 (20 phr, 40 phr, 60 phr), namely the predicted curves of the Yeoh constitutive model that explicitly incorporates the carbon black filling volume fraction, were obtained, as shown in Figure 8. From Figure 8, the predicted curves match well with the experimental curves. This indicates that the Yeoh constitutive model, which explicitly incorporates the carbon-black-filling volume fraction, can accurately describe the nonlinear hyper-elastic mechanical behavior of carbon-black-filled rubber within different ranges of carbon-black-filling volume fractions at 150% strain.

**Figure 9:**
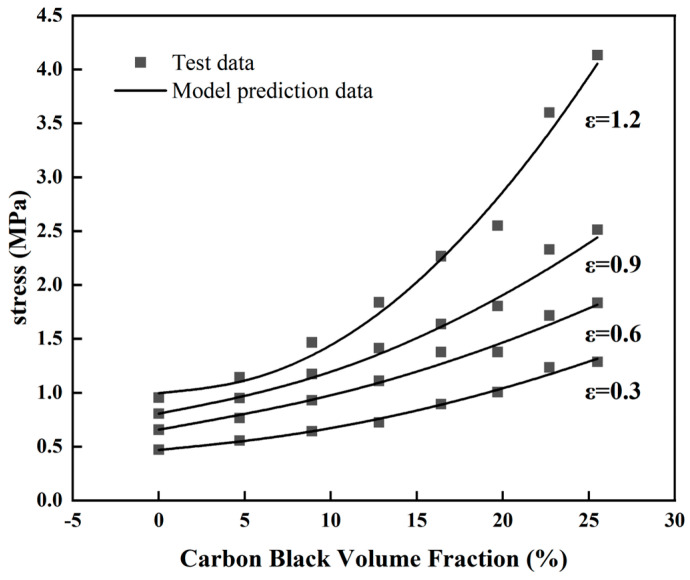
The variation trend of rubber stress with the volume fraction of CB under constant strain and the corresponding model prediction curve (*ε* is strain in the figure).

To further explore the correlation between the hyper-elastic properties of natural rubber and carbon black filling, the characterization parameters in Table 4 were utilized along with the Yeoh constitutive model to predict the stress of the rubber under constant elongation over the carbon-black-filling volume fraction. The predictions of stress at constant elongation are depicted in Figure 9. From Figure 9, it can be observed that the Yeoh constitutive model, which explicitly characterizes the carbon-black-filling volume fraction, accurately captures the stress enhancement phenomenon under different levels of carbon-black-filling volume fractions and different constant elongation ratios. Specifically, as the carbon-black-filling volume fraction increases, the stress in natural rubber consistently and nonlinearly increases. This indicates that the carbon black filling progressively reinforces (effectively “hardens”) the natural rubber, and the degree of reinforcement increases with the higher volume fraction of carbon black filling. In practical industrial applications where certain rubber formulations are costlier to test, after the mechanical property tests on rubber samples within a defined range of carbon black filling, the Yeoh constitutive model that explicitly characterizes the volume fraction of carbon black filling can be used to predict the performance of other rubber formulations within a certain deformation range. This approach offers a practical and viable method for real-world applications.

## 5. Conclusions

By means of the Yeoh constitutive model based on the continuum mechanics theory, the Yeoh constitutive model for explicitly characterizing the volume fraction of carbon black filling was constructed, and the following conclusions can be drawn:

(1) Through uniaxial tensile tests on the natural rubber with varying levels of carbon black filling contents at a significant deformation range (150% elongation), the correlation between the hyper-elastic properties of natural rubber and the carbon black filling was revealed. The stress-strain curves of natural rubber became steeper with an increasing carbon black filling content, indicating that natural rubber gradually becomes “stiffer” as the filling with carbon black increases. The “S”−shaped nonlinearity in the stress-strain curve also became increasingly prominent.
(2) A quantitative relationship between the Yeoh constitutive model and the volume fraction of carbon black filling was established. It explicitly characterizes the correlation between the hyper-elastic mechanical behavior of the rubber material and the carbon black filling. This further extends the applicability of the Yeoh constitutive model and allows for the prediction of the correlation between the hyper-elastic properties of natural rubber and the carbon black filling. During different stages of deformation in the tensile process, the degree of enhancement of natural rubber by the carbon black filling varies. This is reflected in the non-linear change in the stress of the rubber material with increasing tensile strain, showing a pattern of initially decreasing and then increasing over the increase in strain. Furthermore, as the carbon black filling amount increases, the nonlinear change characterized by an initial decrease followed by an increase becomes more pronounced.
(3) The Yeoh constitutive model, which explicitly represents the volume fraction of carbon black filling, accurately captures the stress enhancement phenomenon under different levels of carbon black filling and different constant elongation ratios. This indicates that the effect of the carbon black filling on the performance of natural rubber, essentially making it progressively “stiffer”, is significant. The degree of enhancement increases as the volume fraction of carbon black filling increases. When conducting tests on certain rubber materials with higher costs, once the mechanical performance on rubber samples within a specific range of carbon black filling was determined, the Yeoh constitutive model, which explicitly represents the volume fraction of carbon black filling, could be used to predict the performance of other rubber formulations within a certain range of deformations. This approach offers a highly practical and viable method for real-world applications.

## Figures and Tables

**Table 1:** Reagents used in the experiment.

Material Name	Specification	Manufacturers
NR		Shandong Haoshun Chemical Co., Ltd., Jinan, Shandong, China
CB	N220	Tianjin Zhengning New Materials Co., Ltd., Tianjin, China
Stearic acid		Industrial-grade
Zinc oxide		Industrial-grade
Sulfur		Industrial-grade
Accelerator NS		Industrial-grade
Antioxidant	4020	Industrial-grade

(NR: natural rubber).

**Table 2:** Formulas and codes of eight kinds of carbon-black-filled vulcanized rubber (UNIT: PHR).

Specimen Code	NR	CB N220	Zinc Oxide	Stearic Acid	Sulfur	Accelerator NS	Antioxidant 4020	Total	CB Volume Fraction (%)
NR-0	100	0	5	3	2	1	1.2	112.2	0
NR-1	100	10	5	3	2	1	1.2	122.2	4.7
NR-2	100	20	5	3	2	1	1.2	132.2	8.9
NR-3	100	30	5	3	2	1	1.2	142.2	12.8
NR-4	100	40	5	3	2	1	1.2	152.2	16.4
NR-5	100	50	5	3	2	1	1.2	162.2	19.7
NR-6	100	60	5	3	2	1	1.2	172.2	22.7
NR-7	100	70	5	3	2	1	1.2	182.2	25.5

**Table 3:** Model fitting parameters of rubber with different CB fillings.

CB (phr)	CB (vol.%)	*C*_10_	*C*_20_	*C*_30_	*R* ^2^
0	0	0.30318	−0.02264	0.00254	0.996
10	4.7	0.3521	−0.0262	0.00337	0.992
30	12.8	0.4981	−0.0415	0.008581	0.998
50	19.7	0.6781	−0.09644	0.02217	0.998
70	25.5	0.9073	−0.1609	0.04587	0.999

**Table 4:** Characterization parameters of volume fraction dependence of CB filling.

Model Parameter	Characterization Parameters			*R* ^2^
*C*_10_	*A*_0_ = 0.30622	*A*_1_ = 0.00576	*A*_2_ = 6.91 × 10^−4^	0.99991
*C*_20_	*B*_0_ = −0.02518	*B*_1_ = 0.00225	*B*_2_ = −2.06 × 10^−4^	0.99884
*C*_30_	*C*_0_ = 0.0037	*C*_1_ = −9.12 × 10^−4^	*C*_2_ = 9.90 × 10^−5^	0.99642

## Data Availability

The data presented in this study are available on request from the corresponding author. The data are not publicly available due to that CNKI has not uploaded the relevant master’s thesis yet.

## References

[1] Peng X., Li L. (2020). State of the art of constitutive relations of hyperelastic materials. Chin. J. Theo-Retical Appl. Mech..

[2] Ding F., Zhang H., Ding M. (2019). −M.; Shi, T.F.; Li, Y.Q.; An, L.J. Theoretical Models for Stress-Strain Curves of Elastomer Materials. ACTA Polym. Sin..

[3] Ogden R.W. (1997). Non-Linear Elastic Deformations.

[4] Boyce M.C., Arruda E.M. (2000). Constitutive models of rubber elasticity: A review. Rubber Chem. Technol..

[5] Steinmann P., Hossain M., Possart G. (2012). Hyperelastic models for rubber-like materials: Consistent tangent operators and suitability for Treloar’s data. Arch. Appl. Mech..

[6] Koprowski-Theiss N., Johlitz M., Diebels S. (2011). Characterizing the time dependence of filled EPDM. Rubber Chem. Technol..

[7] Mansouri M., Darijani H. (2014). Constitutive modeling of isotropic hyperelastic materials in an exponential framework using a self-contained approach. Int. J. Solids Struct..

[8] Zhao Z., Mu X., Du F. (2019). Modeling and verification of a new hyperelastic model for rubber-like materials. Math. Probl. Eng..

[9] Poomuthu A., Stoček R., Chattopadhyay S., Khastgir D., Kaliske M., Özenç K., Sekar P. (2020). Understanding fracture of a carbon black filled rubber compound using material force theory. Theor. Appl. Fract. Mech..

[10] El Yaagoubi M., Meier J., Juhre D. (2020). Lifetime prediction of carbon black filled elastomers based on the probability distribution of particle using an inelastic and hyperelastic material model. Eng. Fail. Anal..

[11] Gudsoorkar U., Bindu R. (2021). Computer simulation of hyper elastic re-treaded tire rubber with ABAQUS. Mater. Today Proc..

[12] He H., Zhang Q., Zhang Y., Chen J., Zhang L., Li F. (2022). A comparative study of 85 hyperelastic constitutive models for both unfilled rubber and highly filled rubber nanocomposite material. Nano Mater. Sci..

[13] Arruda E.M., Boyce M.C. (1993). A three-dimensional constitutive model for the large stretch behavior of rubber elastic materials. J. Mech. Phys. Solids.

[14] James H.M., Guth E. (1943). Theory of the elastic properties of rubber. J. Chem. Phys..

[15] Yeoh O.H. (1993). Some forms of the strain energy function for rubber. Rubber Chem. Technol..

[16] Mooney M. (1940). A theory of large elastic deformation. J. Appl. Phys..

[17] Yeoh O.H. (1990). Characterization of elastic properties of carbon-black-filled rubber vulcanizates. Rubber Chem. Technol..

[18] Rivlin R.S. (1948). Large elastic deformations of isotropic materials. I. Fundamental concepts. Philos. Trans. R. Soc. Lond. Ser. A-Math. Phys. Sci..

[19] (2014). Rubber-Measurement of Vulcanization Characteristics with the Oscillating Disc Curemeter.

[20] (2017). Rubber, Vulcanized or Thermoplastic—Determination of Tensile Stress-Strain Properties.

[21] Fukahori Y. (2003). The mechanics and mechanism of the carbon black reinforcement of elastomers. Rubber Chem. Technol..

